# Prevalence and prognosis of tinnitus in post-COVID-19 patients: a cross-sectional survey

**DOI:** 10.1017/S095026882400147X

**Published:** 2024-11-08

**Authors:** Shihang Mao, Dantong Gu, Di Wang, Peifan Li, Xiaoling Huang, Haoning Yin, Shan Sun

**Affiliations:** 1 NHC Key Laboratory of Hearing Medicine (Fudan University), Demin Han’s Academician Workstation, Eye & ENT Hospital, Otolaryngology Research Institute, Shanghai, PR China; 2 Clinical Research Unit of Eye & ENT Hospital, Fudan University, Shanghai, PR China; 3 No. 2 High School of East China Normal University, Shanghai, PR China

**Keywords:** post-COVID-19, prognosis, severity, tinnitus, treatment outcomes

## Abstract

Recent developments have indicated a potential association between tinnitus and COVID-19. The study aimed to understand tinnitus following COVID-19 by examining its severity, recovery prospects, and connection to other lasting COVID-19 effects. Involving 1331 former COVID-19 patients, the online survey assessed tinnitus severity, cognitive issues, and medical background. Of the participants, 27.9% reported tinnitus after infection. Findings showed that as tinnitus severity increased, the chances of natural recovery fell, with more individuals experiencing ongoing symptoms (*p <* 0.001). Those with the Grade II mild tinnitus (OR = 3.68; CI = 1.89–7.32; *p* = 0.002), Grade III tinnitus (OR = 3.70; CI = 1.94–7.22; *p* < 0.001), Grade IV (OR = 6.83; CI = 3.73–12.91; *p* < 0.001), and a history of tinnitus (OR = 1.96; CI = 1.08–3.64; *p* = 0.03) had poorer recovery outcomes. Grade IV cases were most common (33.2%), and severe tinnitus was strongly associated with the risk of developing long-term hearing loss, anxiety, and emotional disorders (*p* < 0.001). The study concludes that severe post-COVID tinnitus correlates with a worse prognosis and potential hearing loss, suggesting the need for attentive treatment and management of severe cases.

## Introduction

Tinnitus, characterized by the perception of noise or ringing in the ears, is a prevalent symptom affecting approximately 10–15% of the global population [1]. Although it can arise from various underlying conditions, recent developments have indicated a potential association between tinnitus and COVID-19, the disease caused by the novel coronavirus SARS-CoV-2. COVID-19 first emerged in late 2019 and rapidly escalated into a global pandemic, impacting millions of individuals worldwide. Although the virus primarily affects the respiratory system, it has also been linked to several other manifestations [2], some experienced tinnitus during or after the infection [3–6]. Dizziness [7] and notably tinnitus [8–11] have been identified as common symptoms of COVID-19 [12], persisting for a significant duration, leading to long-term COVID [10, 13], a multisystemic condition encompassing severe symptoms that develop during or following COVID-19 infection. Long-term COVID can cause more than 200 kinds of different symptoms and the time of recovery exceeds 35 weeks [14].

Although the exact relationship between tinnitus and COVID-19 is still under investigation, several theories have emerged. Some researchers suggest that the virus may induce inflammation in the auditory system [15–17], while others propose that the stress and anxiety associated with the pandemic may exacerbate existing tinnitus or contribute to its onset in predisposed individuals [18].

Although current studies on post-COVID-19 tinnitus have predominantly focused on symptomatology and duration, less attention has been given to prognosis, which is a critical concern for patients. Persistent symptoms can significantly impact patients’ lives and potentially give rise to various health issues. To gain a better understanding of the progression and prognosis of post-COVID-19 tinnitus, our study investigated the severity and prognosis in patients recovering from COVID-19 using an online survey. Various methods have been employed in different studies to assess the impact of tinnitus, including measures of tinnitus severity, bother, annoyance, sleep disruption, and concentration.

This article provides a comprehensive overview of tinnitus and its potential association with COVID-19, discusses the current state of research, and provides possible explanations for this association and implications for treatment and management. It is important to note that our understanding of COVID-19 is continuously evolving, and further research is needed to fully comprehend the relationship between COVID-19 and tinnitus.

## Materials and methods

### Participants

Using convenience sampling, which makes people who have access to the questionnaire part of the study as participants to recruit participants, this study included 1366 participants who had tested positive for COVID-19. To ensure data quality, certain exclusion criteria were applied. Participants who provided inconsistent answers if they reported symptoms but did not provide any information on prognosis or if they indicated no symptoms yet reported receiving treatment for tinnitus, omitted vital details such as gender, age, height, weight, or infection date, and those under the age of 18 were excluded from the study. After applying these criteria, a final sample of 1331 participants was included in the study, of which 371 individuals reported experiencing post-COVID-19 tinnitus.

All participants in the study were confirmed to have contracted COVID-19 through nucleic acid or antigen tests conducted during the pandemic period, specifically from 1 December 2022 to 31 January 2023. We specially instructed participants to provide the date of their first COVID-19 infection. Participants were residents of various provinces in China who were recruited through an online survey.

The online questionnaire was administered nationwide from 19 January 2023 to 11 February 2023. Ethical considerations were strictly followed in accordance with the Measures for the Ethical Review of Biomedical Research Involving Humans of the National Health Commission of the People’s Republic of China. The research protocol received ethical approval from the Eye and ENT Hospital Ethics Panel at Fudan University (Approval No. 2022127) for international data collection.

### Outcomes

#### Primary outcome

**Figure 1.**
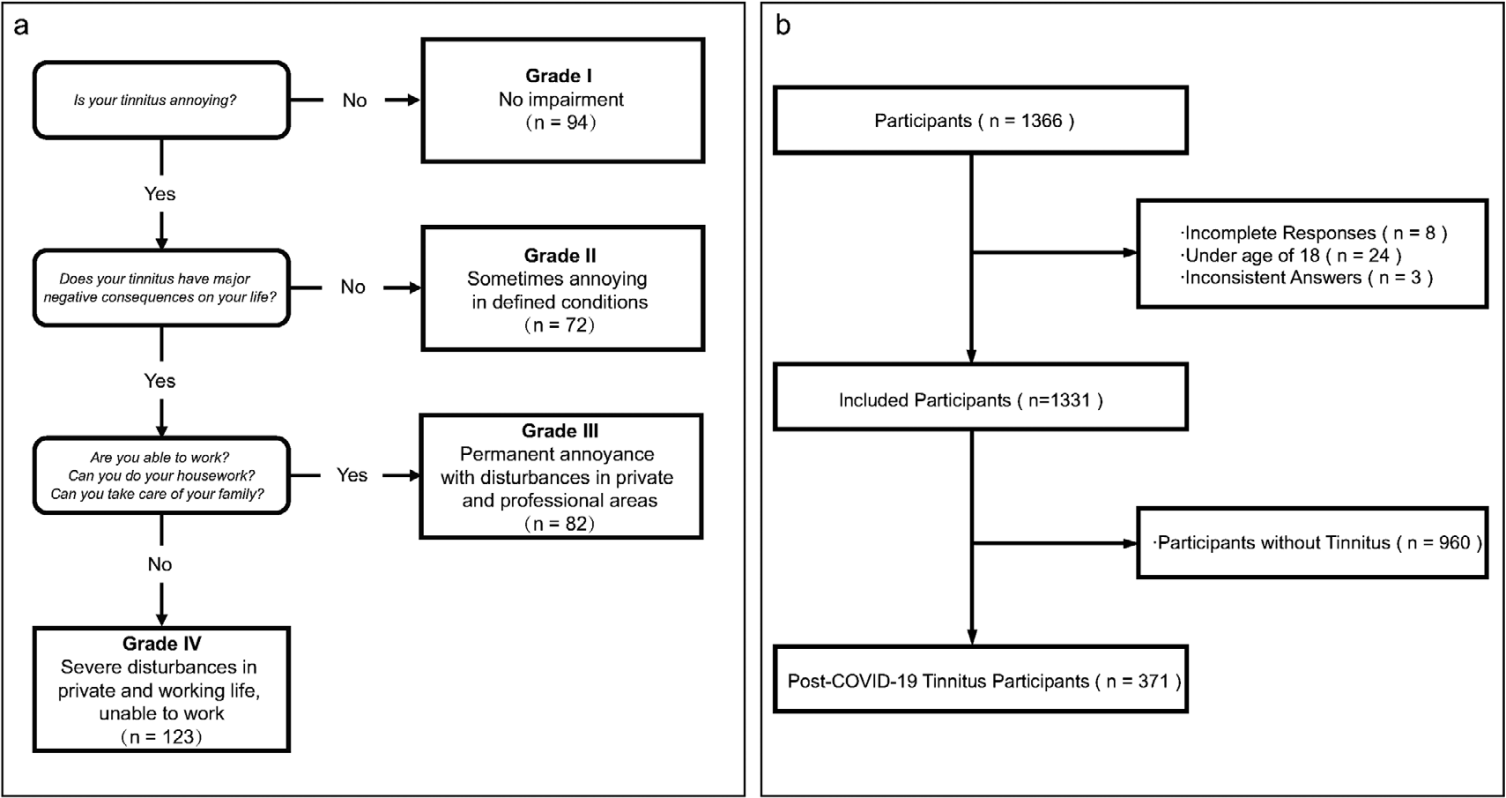
Tinnitus questionnaire of Biesinger and flow chart of included post-COVID-19 tinnitus participant. (a) Tinnitus questionnaire of Biesinger: assessment of tinnitus severity (modified and translated from Biesinger and colleagues) and number of each tinnitus severity grade. (b) Flow chart of included post-COVID-19 tinnitus participant: flow path showing the process of excluding 35 participants consisting of 8 with incomplete questionnaire responses, 24 under the age of 18, and 3 with inconsistent answers.

The primary objective of this study was to determine the prevalence of tinnitus among participants and the prognosis of tinnitus. Tinnitus severity was assessed using the tinnitus severity questionnaire of Biesinger, which is a pragmatic classification based on clinical situations brought up by German scientists correlating well with the tinnitus questionnaire of Goebel and Hiller (Figure1a) [19, 20]. The questionnaire consists of three questions dividing participants into four grades of tinnitus severity. For prognosis, participants were requested to classify it as untreated and self-healed, full remission, relief, or failure.

#### Secondary outcomes

The study also assessed cognitive impairment and emotional status using the cognition and emotion vectors in Health Utilities Index Mark 3 [21]. Participants were categorized into six levels of cognitive impairment and five levels of emotional status. We used the Generalized Anxiety Disorder 2-item and Patient Health Questionnaire-2 to evaluate anxiety and depression. Scores under 3 refer to no anxiety or depression, whereas scores from 3 to 6 refer to anxiety or depression status according to each scale.

Additionally, the study examined pre-existing medical conditions related to the ear, nose, and throat (ENT), including tinnitus, loss of hearing, loss of taste, and loss of smell, prior to the onset of the new coronavirus infection.

### Study design

We employed a cross-sectional research design using an anonymous online questionnaire. The questionnaire was developed collaboratively by two experienced doctors specializing in otolaryngology and public health. It consisted of informed consent, and 52 questions including basic patient information, date of SARS-CoV-2 infection, tinnitus symptoms and outcomes, pre-existing ENT medical history, and underlying medical conditions, utilizing formats such as single-choice, multiple-choice, and self-filling (Supplementary material 1). For more detailed information about the questionnaire, please visit the following link: https://www.wjx.cn/vm/exOmDok.aspx.

The study was conducted in accordance with the principles of the Declaration of Helsinki and was approved by the Clinical Research Ethics Committee of the Eye & ENT Hospital of Fudan University. All collected data were managed anonymously to ensure confidentiality. Data collection was carried out through self-administration of electronic questionnaires.

### Statistical analysis

Non-normally distributed continuous variables were described using the median and interquartile range, and their comparison was performed using the Wilcoxon rank sum test. Categorical variables were calculated as frequencies and percentages and were analysed using the chi-square test or Fisher’s exact test, as appropriate. Univariate logistic regression was performed to evaluate the relationship between treatment effect and potential variables. Multivariate logistic regression was used to identify independent predictors of treatment effect, and a forest plot was generated. The relative risks at increasing cumulative exposures were calculated and used to estimate the absolute risk reduction. Statistical significance was defined as *p* < 0.05 for all analyses. R Studio (version 2023.06.1+524), was used for all statistical analyses and model performance evaluation.

## Results

### Participant characteristics

**Figure 2.**
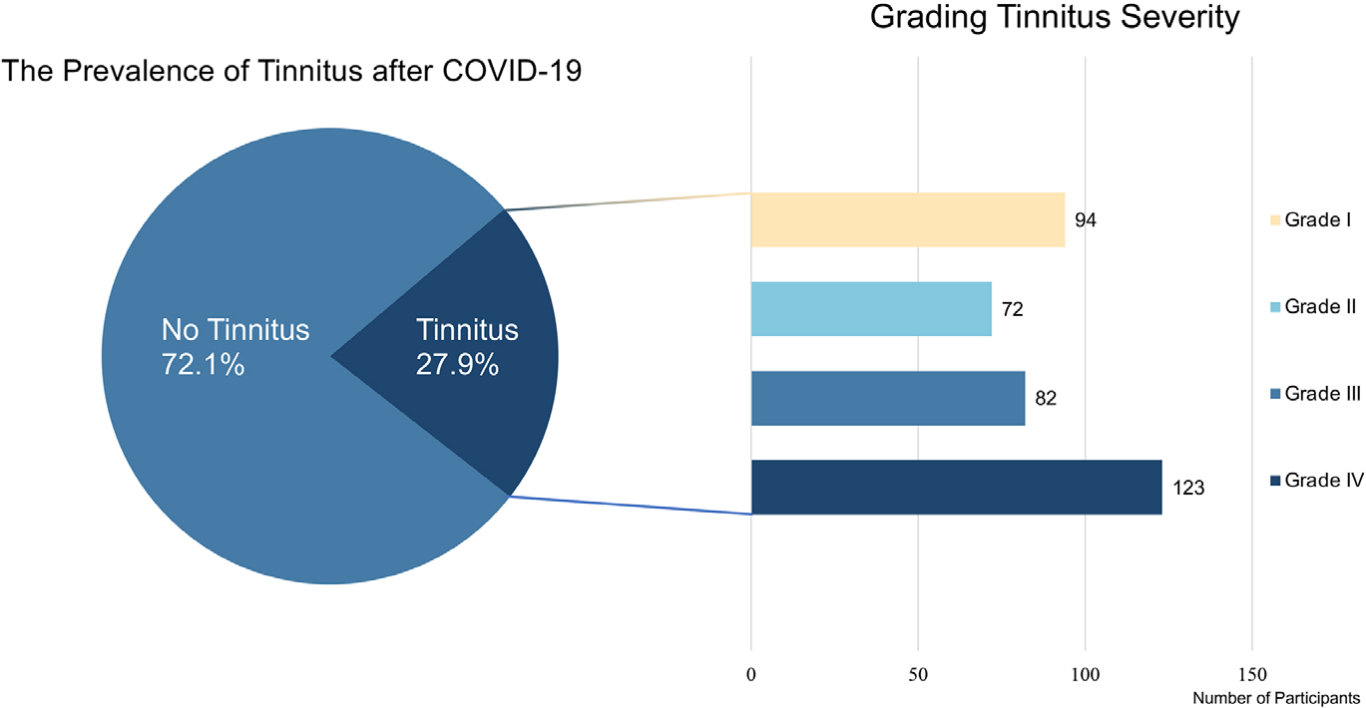
Prevalence of tinnitus in the post-COVID-19 population and severity grading. This figure displays the prevalence of tinnitus among participants in the post-COVID-19 population that was surveyed. Tinnitus patients were categorized into four groups based on tinnitus severity, namely Grade I (slight tinnitus), Grade II (mild tinnitus), Grade III (moderate tinnitus), and Grade IV (severe tinnitus). The left panel of the figure represents the number of participants along the X-axis.

A total of 1366 individuals participated in this study, but only 1331 participants were included in the final analysis due to 8 incomplete questionnaire responses, 24 under the age of 18, and 3 with inconsistent answers (Figure 1b). Among the 1331 participants, 371 individuals developed tinnitus symptoms following COVID-19 infection, resulting in a prevalence rate of 27.9% (Figure 2). The study population consisted of 18% males and 82% females, with a median age of 34 years (interquartile range, 28–42 years). Tinnitus was reported in both ears by 51.2%. The majority of participants had a high level of education, with 66.3% having an undergraduate degree and 25.9% having a graduate or higher degree. The most common comorbidities, aside from ENT symptoms, were hypertension (5.7%) and diabetes (2.2%), which were believed to influence tinnitus (Table 1).

**Table 1.** Demographic characteristics of participants with post-COVID-19 tinnitus

Characteristics		Overall (*N* = 371)	Male (*N* = 67, 18%)	Female (*N* = 304, 82%)	*p-*Value	SMD
Age, median [interquartile]		34.00 [28.00, 42.00]	38.00 [28.00, 46.00]	34.00 [28.00, 41.00]	0.067	0.311
Height, median [interquartile]		163.00 [160.00, 169.00]	176.00 [170.50, 178.50]	162.00 [159.00, 166.00]	**<0.001**	2.212
Weight, median [interquartile]		60.00 [53.00, 70.20]	78.00 [67.50, 85.00]	58.00 [52.00, 65.00]	**<0.001**	0.868
BMI, median [interquartile]		22.50 [20.30, 25.49]	25.11 [22.94, 27.00]	21.98 [20.02, 24.81]	**<0.001**	0.275
Education, *n* (%)	Doctor	21 (5.7)	7 (10.4)	14 (4.6)	0.272	0.294
	Master	75 (20.2)	11 (16.4)	64 (21.1)		
	Undergraduate	246 (66.3)	42 (62.7)	204 (67.1)		
	High school	22 (5.9)	5 (7.5)	17 (5.6)		
	Junior high school	6 (1.6)	2 (3.0)	4 (1.3)		
	Other	1 (0.3)	0 (0.0)	1 (0.3)		
City, *n* (%)	Shanghai	86 (23.2)	22 (32.8)	64 (21.1)	0.056	0.268
	Other	285 (76.8)	45 (67.2)	240 (78.9)		
Position, *n* (%)	Left	97 (26.2)	20 (29.8)	77 (25.3)	0.354	0.195
	Right	84 (22.6)	18 (26.9)	66 (21.7)		
	Bilateral	190 (51.2)	29 (43.3)	161 (53.0)		
Severity, *n* (%)	Grade I-slight	94 (25.3)	12 (17.9)	82 (27.0)	**0.027**	0.421
	Grade II-mild	72 (19.4)	15 (22.4)	57 (18.7)		
	Grade III-moderate	82 (22.1)	9 (13.4)	73 (24.0)		
	Grade IV-severe	123 (33.2)	31 (46.3)	92 (30.3)		
Prognosis, *n* (%)	Self-healed	151 (40.7)	20 (29.9)	131 (43.1)	0.133	0.307
	Full remission	12 (3.2)	2 (3.0)	10 (3.3)		
	Relief	24 (6.5)	7 (10.4)	17 (5.6)		
	Failure	184 (49.6)	38 (56.7)	146 (48.0)		
History, *n* (%)	Yes	62 (16.7)	13 (19.4)	49 (16.1)	0.637	0.086
	No	309 (83.3)	54 (80.6)	255 (83.9)		
Hypertension, *n* (%)	Yes	21 (5.7)	7 (10.4)	14 (4.6)	0.114	0.223
	No	350 (94.3)	60 (89.6)	290 (95.4)		
Diabetes, *n* (%)	Yes	8 (2.2)	1 (1.5)	7 (2.3)	1.000	0.059
	No	363 (97.8)	66 (98.5)	297 (97.7)		

Based on the tinnitus severity grade, the 371 patients were categorized into the following four levels of severity: Grade I-slight tinnitus (25.3%), Grade II-mild tinnitus (19.4%), Grade III-moderate tinnitus (22.1%), and Grade IV-severe tinnitus (33.2%). Of the participants, 16.7% had a previous history of tinnitus (Figure 2).

After conducting statistical analysis, we found a significant difference only in the height, weight, and body mass index of the patients. No significant differences were observed among other demographic variables and comorbidities (Table 1).

### Severity grading and prognosis of post-COVID-19

In assessing the prognosis of tinnitus after COVID-19, our primary focus was on evaluating changes in patients’ symptoms.

Among the 371 patients reporting tinnitus included in the study, 151 experienced spontaneous recovery without any treatment, 12 were in full remission, 24 experienced relief, and 184 had no recovery following treatment (Table 2).

**Table 2.** Correlation between treatment effect of post-COVID-19 tinnitus and potential variables

Characteristics	Level	Overall (*N* = 371)	Self-healed (*N* = 151)	Full remission (*N* = 12)	Relief (*N* = 24)	Failure (*N* = 184)	*p-*Value	SMD
Age, median [interquartile]		34.00 [28.00, 42.00]	34.00 [27.00, 40.00]	39.00 [32.50, 43.50]	33.50 [31.00, 42.50]	36.00 [28.00, 44.00]	0.073	0.295
Height, median [interquartile]		163.00 [160.00, 169.00]	163.00 [159.00, 169.50]	160.00 [159.00, 167.00]	165.00 [158.75, 170.50]	163.50 [160.00, 168.00]	0.725	0.184
Weight median [interquartile]		60.00 [53.00, 70.20]	60.00 [52.00, 70.20]	59.50 [53.50, 76.00]	60.00 [54.75, 76.25]	60.50 [54.75, 70.00]	0.599	0.111
BMI, median [interquartile]		22.50 [20.30, 25.49]	22.06 [20.03, 25.50]	23.24 [20.82, 25.30]	23.12 [20.22, 25.50]	22.59 [20.57, 25.42]	0.714	0.085
Education, *n* (%)	Doctor	21 (5.7)	11 (7.3)	0 (0.0)	2 (8.3)	8 (4.3)		
	Master	75 (20.2)	30 (19.9)	3 (25.0)	4 (16.7)	38 (20.7)		
	Undergraduate	246 (66.3)	101 (66.9)	8 (66.7)	17 (70.8)	120 (65.2)		
	Junior high school	6 (1.6)	0 (0.0)	0 (0.0)	0 (0.0)	6 (3.3)		
	High school	22 (5.9)	9 (5.9)	1 (8.3)	1 (4.2)	11 (6.0)		
	Other	1 (0.3)	0 (0.0)	0 (0.0)	0 (0.0)	1 (0.5)		
City, *n* (%)	Shanghai	86 (23.2)	32 (21.2)	2 (16.7)	9 (37.5)	43 (23.4)	0.352	0.249
	Other	285 (76.8)	119 (78.8)	10 (83.3)	15 (62.5)	141 (76.6)		
Position, *n* (%)	Left	97 (26.2)	37 (24.5)	4 (33.3)	6 (25.0)	50 (27.2)	0.892	0.165
	Right	84 (22.6)	39 (25.8)	3 (25.0)	5 (20.8)	37 (20.1)		
	Bilateral	190 (51.2)	75 (49.7)	5 (41.7)	13 (54.2)	97 (52.7)		
Severity, *n* (%)	Grade I-slight	94 (25.3)	72 (47.7)	0 (0.0)	1 (4.2)	21 (11.4)	**<0.001**	1.075
	Grade II-mild	72 (19.4)	32 (21.2)	1 (8.3)	2 (8.3)	37 (20.1)		
	Grade III-moderate	82 (22.1)	32 (21.2)	2 (16.7)	5 (20.8)	43 (23.4)		
	Grade IV-severe	123 (33.2)	15 (9.9)	9 (75.0)	16 (66.7)	83 (45.1)		
History	Yes	62 (16.7)	18 (11.9)	0 (0.0)	3 (12.5)	41 (22.3)	**0.028**	0.395
	No	309 (83.3)	133 (88.1)	12 (100.0)	21 (87.5)	143 (77.7)		
Hypertension	Yes	21 (5.7)	6 (4.0)	2 (16.7)	0 (0.0)	13 (7.1)	0.131	0.362
	No	350 (94.3)	145 (96.0)	10 (83.3)	24 (100.0)	171 (92.9)		
Diabetes	Yes	8 (2.2)	3 (2.0)	1 (8.3)	0 (0.0)	4 (2.2)	0.434	0.237
	No	363 (97.8)	148 (98.0)	11 (91.7)	24 (100.0)	180 (97.8)		

**Figure 3.**
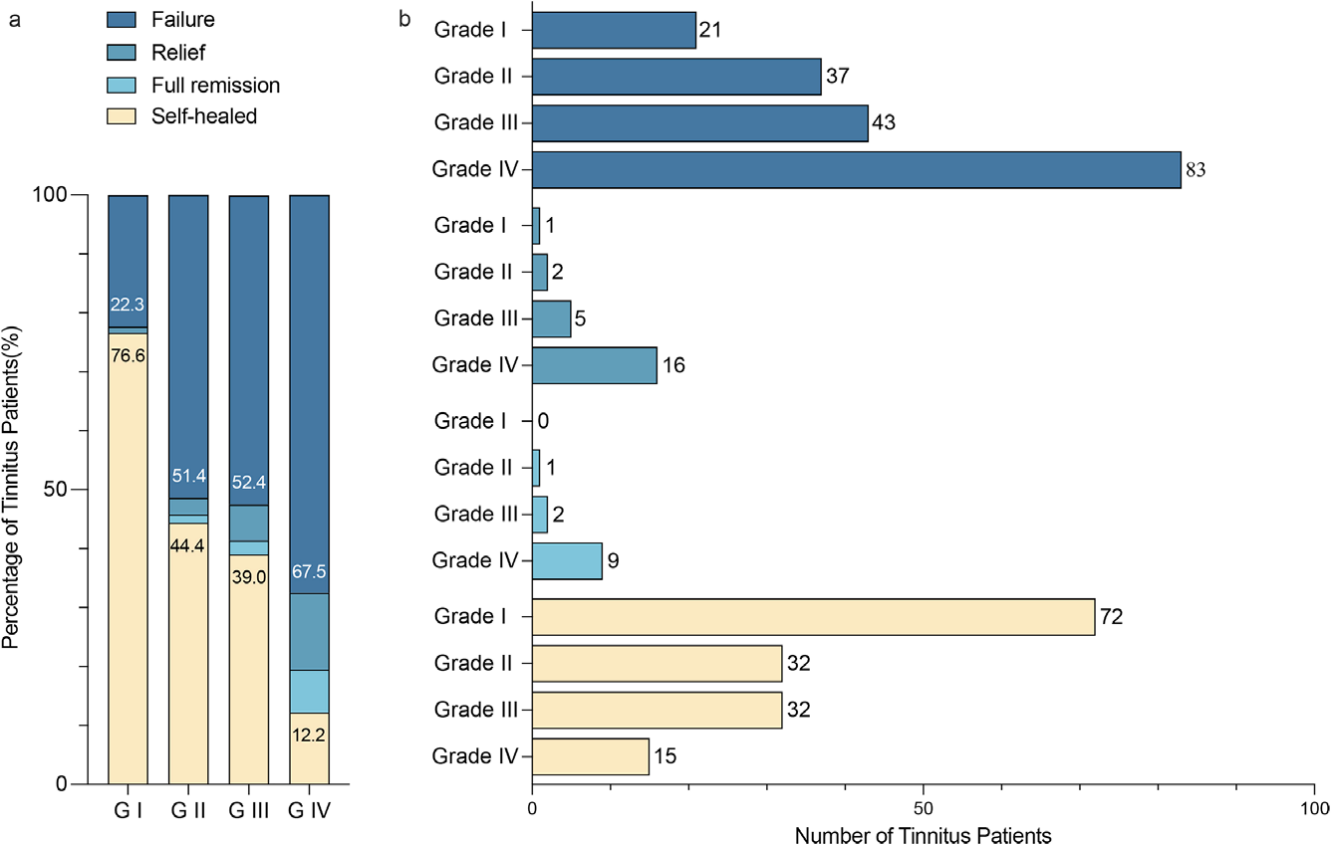
Tinnitus prognosis in post-COVID-19 patients. (a) This panel shows the percentage of tinnitus patients distributed across various severity grades. (b) This panel represents the number of tinnitus patients within each severity grade. It is evident that more severe cases of tinnitus exhibit lower rates of self-healing and recovery.

We observed variations in the prognosis of patients based on their tinnitus severity. As the severity grade increased, the rate of self-recovery in patients with tinnitus after COVID-19 gradually decreased, while the number of patients with persistent tinnitus symptoms increased (Figure 3a). Among the 94 patients with slight tinnitus after COVID-19, 72 (76.6%) experienced spontaneous recovery without treatment, no patient achieved full remission after tinnitus-related treatment, and 1 (1.1%) experienced symptom relief following treatment. However, 21 (22.3%) patients continued to experience persistent tinnitus symptoms despite treatment. Among the 72 patients with mild tinnitus, 32 (44.4%) experienced spontaneous recovery, 1 (1.4%) achieved full remission after tinnitus-related treatment, and 2 (2.8%) experienced symptom relief. However, 37 (51.4%) continued to experience long-term tinnitus symptoms after treatment. For the 82 patients with moderate tinnitus, 32 (39.0%) experienced spontaneous recovery, 2 (2.4%) achieved full remission after tinnitus-related treatment, 5 (6.1%) experienced partial relief following treatment, and 43 (52.4%) continued to experience long-term tinnitus symptoms after treatment. Among the 123 patients with severe tinnitus, 15 (12.2%) experienced self-recovery from tinnitus, 9 (7.3%) achieved full remission after tinnitus-related treatment, and 16 (13.0%) experienced partial relief after treatment. However, 83 (67.5%) still had the same persistent tinnitus symptoms despite treatment (Figure 3b).

Except for the severity grade, our analysis did not find any significant associations between the prognosis of tinnitus and factors (Table 2).

### Association of long-term COVID-19 symptoms and tinnitus severity

**Figure 4.**
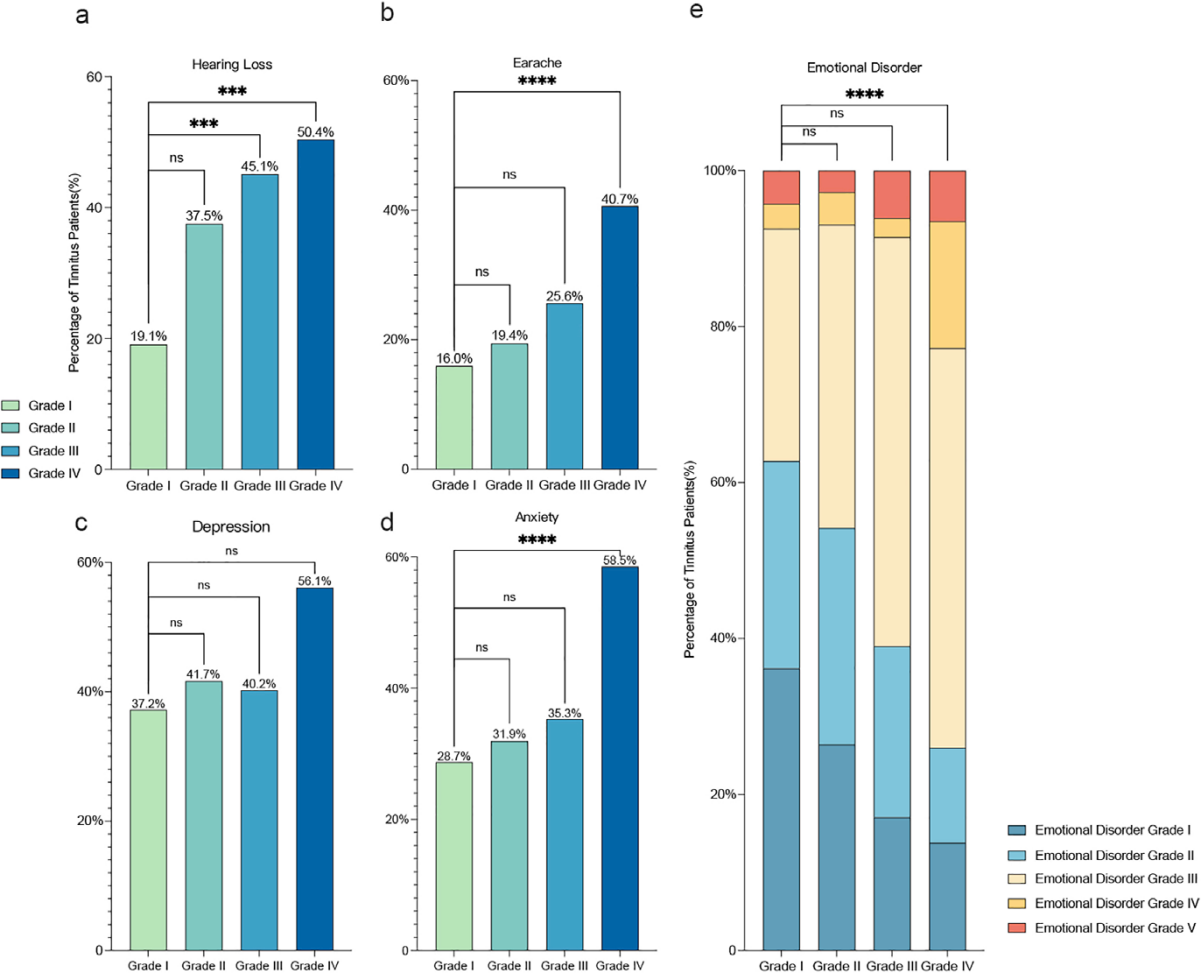
Associations between tinnitus severity and long-term COVID-19 symptoms. (a) Morbidity rate of hearing loss in post-COVID-19 tinnitus patients with different severity grades. The percentages of tinnitus patients experiencing hearing loss symptoms categorized into different severity grades are shown. The severity grades represent the extent of hearing loss in relation to tinnitus. Notably, patients with Grades III and IV exhibited a significantly higher risk of developing hearing loss. (b) Morbidity rate of earache in post-COVID-19 tinnitus patients with different severity grades. The percentages of tinnitus patients experiencing earache symptoms categorized into different severity grades are shown. Notably, patients with Grade IV exhibited a significantly higher risk of suffering from earache. (c) Morbidity rate of depression in post-COVID-19 tinnitus patients with different severity grades. The percentages of tinnitus patients experiencing depression categorized into different severity grades are shown. The severity grades represent the extent of depression in relation to tinnitus. There is no significant difference. (d) Morbidity rate of anxiety in post-COVID-19 tinnitus patients with different severity grades. The percentages of tinnitus patients experiencing anxiety categorized into different severity grades are shown. The severity grades represent the extent of hearing loss in relation to tinnitus. Notably, patients with Grade IV exhibited a significantly higher risk of developing hearing loss. (e) Morbidity rate of emotional disorder in post-COVID-19 tinnitus patients with different severity grades. The percentages of tinnitus patients experiencing emotional disorders categorized into different severity grades are shown. Notably, patients with Grade IV exhibited a significantly higher risk of emotional disorder.

The severity of tinnitus may be affected by other long-term COVID-19 symptoms, including otological symptoms, hyposmia, and hypogeusia, which are the most typical and bothersome associated with tinnitus. A chi-square analysis of pairwise comparisons among the four groups of participants with different tinnitus severity levels revealed a significant relationship between severity grade and the incidence of earache and hearing loss (*p* < 0.001). The morbidity rate of hearing loss in patients with tinnitus after COVID-19 gradually increased along the severity grade from 19.1% to 50.4%. Grade III (*p* < 0.001) and Grade IV (*p* < 0.001) tinnitus significantly contributed to the risk of hearing loss (Figure 4a). Patients with Grade IV tinnitus had a higher risk of earache (*p* < 0.0001) (Figure 4b).

The morbidity rates for loss of smell (*p* = 0.274, SMD = 0.143) and loss of taste (*p* = 0.191, SMD = 0.169) did not show significant differences among patients with distinct tinnitus severity levels.

Psychological status is one of the most common and painful symptoms of long-term COVID-19 syndrome. There are strong correlations between the severity of tinnitus and psychological status, including cognitive impairment, emotional disorder, anxiety, and depression in the general population. Therefore, tinnitus after COVID-19 may also lead to, or be affected by certain related obstacles. Participants were categorized into six levels based on their cognitive status and five levels for emotional disorder. Approximately 28.0% of the participants did not experience any cognitive impairment during or after their COVID-19 infection (referred to as grade 1 – no cognitive impairment). The remaining participants reported varying degrees of forgetfulness, confusion, and difficulty in resolving disputes. There was no significant difference in cognitive function among individuals with different severity grades of tinnitus (*p* = 0.517, SMD = 0.320). Anxiety (*p* < 0.001, SMD = 0.325) and emotion (*p* < 0.001, SMD = 0.536), instead, had a relation with post-COVID-19 tinnitus (Table 3). Compared with Grade I tinnitus, Grade IV tinnitus significantly increases the risk of anxiety (*p* < 0.0001) or emotional disorder (*p* < 0.0001) (Figure 4c–e).

**Table 3.** Prevalence of long-term COVID-19 symptoms among participants with post-COVID-19 tinnitus

Characteristics	Tinnitus level	Overall (*N* = 371)	Grade I-slight (*N* = 94, 42.2%)	Grade II-mild (*N* = 72, 20.4%)	Grade III-moderate (*N* = 82, 15.1%)	Grade IV-severe (*N* = 123, 22.3%)	*p-*Value	SMD
Cognition, *n* (%)	Grade1	104 (28.0)	27 (28.7)	22 (30.6)	21 (25.6)	34 (27.6)	0.517	0.320
	Grade 2	58 (15.6)	17 (18.1)	5 (6.9)	12 (14.6)	24 (19.5)		
	Grade 3	133 (35.8)	37 (39.4)	29 (40.3)	30 (36.6)	37 (30.1)		
	Grade 4	64 (17.3)	12 (12.7)	14 (19.4)	15 (18.3)	23 (18.7)		
	Grade 5	11 (3.0)	1 (1.1)	2 (2.8)	3 (3.7)	5 (4.1)		
	Grade 6	1 (0.3)	0 (0.0)	0 (0.0)	1 (1.2)	0 (0.0)		
Emotion, *n* (%)	Grade1	84 (22.6)	34 (40.5)	19 (22.6)	14 (16.7)	17 (20.2)	**<0.001**	0.536
	Grade 2	78 (21.0)	25 (32.1)	20 (25.6)	18 (23.1)	15 (19.2)		
	Grade 3	162 (43.7)	28 (17.3)	28 (17.3)	43 (26.5)	63 (38.9)		
	Grade 4	28 (7.6)	3 (10.7)	3 (10.7)	2 (7.1)	20 (71.5)		
	Grade 5	19(5.1)	4 (21.1)	2 (10.5)	5 (26.3)	8 (42.1)		
Anxiety, *n* (%)	Yes	151(40.7)	27 (17.9)	23 (15.2)	29 (19.2)	72 (47.7)	**<0.001**	0.325
	No	220(59.3)	67 (30.4)	49 (22.3)	53 (24.1)	51 (23.2)		
Depression, *n* (%)	Yes	167(45.0)	35 (20.9)	30 (18.0)	33 (19.8)	69 (41.3)	**0.024**	0.197
	No	204(55.0)	59 (28.9)	42 (20.6)	49 (24.0)	54 (26.5)		
Loss of hearing, *n* (%)	Yes	144 (38.8)	18 (19.1)	27 (37.5)	37 (45.1)	62 (50.4)	**<0.001**	0.369
	No	227 (61.2)	76 (80.9)	45 (62.5)	45 (54.9)	61 (49.6)		
Loss of smell, *n* (%)	Yes	220 (59.3)	56 (59.6)	45 (62.5)	54 (65.9)	65 (52.8)	0.274	0.143
	No	151 (40.7)	38 (40.4)	27 (37.5)	28 (34.1)	58 (47.2)		
Loss of taste, *n* (%)	Yes	200 (53.9)	46 (48.9)	35 (48.6)	52 (63.4)	67 (54.5)	0.191	0.169
	No	171 (46.1)	48 (51.1)	37 (51.4)	30 (36.6)	56 (45.5)		

### Correlation regression analysis between severity grading and prognosis of post-COVID-19 tinnitus

**Figure 5.**
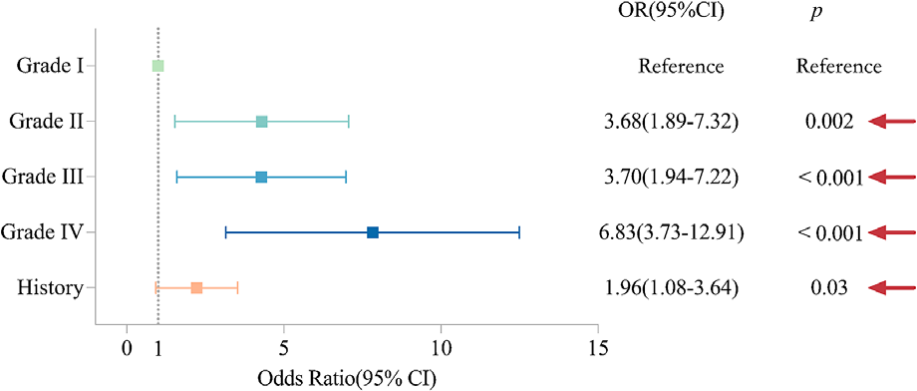
Tinnitus grades and prognosis. Random forest plots for tinnitus patient grading, age, and medical history. Patients with severe tinnitus are associated with less effective treatment outcomes. Grade I tinnitus serves as the reference category. The red arrows indicate statistically significant differences.

We conducted a correlation regression analysis to assess the effectiveness of tinnitus treatment after COVID-19. Our findings indicate that compared to patients with slight Grade I tinnitus, patients with mild tinnitus (OR = 3.68; CI = 1.89–7.32; *p* = 0.002), patients with moderate tinnitus (OR = 3.70; CI = 1.94–7.22; *p* < 0.001), and patients with severe tinnitus (OR = 6.83; CI = 3.73–12.91; *p* < 0.001) all had less favourable treatment outcomes (Figure 5). This finding implies that patients with severe tinnitus were more likely to experience no improvement following treatment, indicating a worse prognosis compared to patients with slight tinnitus. Patients with a history of tinnitus were more likely to experience tinnitus following a COVID-19 infection compared to those without a prior history (OR = 1.96; CI = 1.08–3.64; *p* = 0.03).

## Discussion

Tinnitus is a persistent and bothersome medical symptom commonly associated with long-lasting effects in patients, including those who have experienced long-term COVID. The global general population has shown a morbidity rate of approximately 14% for tinnitus [1, 22]. In China, the prevalence of tinnitus varies among regions, ranging from 10.4% to 14.5% [23, 24]. In our study, the prevalence rate of post-COVID-19 tinnitus was found to be 27.9%, which is higher than that in the general population. This elevated prevalence may be attributed to the ear-related toxicity caused by the novel coronavirus.

Numerous studies have been conducted on COVID-19-associated tinnitus since the outbreak of the pandemic. However, due to variations in medical conditions, the progression of COVID-19, tinnitus evaluation criteria, data collection standards, and other factors, the prevalence rate of tinnitus reported in these studies shows wide variations, with some studies reporting rates as high as 67% [25]. A cross-country survey revealed that the prevalence of tinnitus increased from 11.5% in the first week to 26.2% in respondents with symptoms lasting over 6 months [14]. This suggests that tinnitus symptoms become more common and severe during the progression of COVID-19 and may persist for an extended period during and after the infection. However, a study conducted in Turkey reported that tinnitus gradually alleviated and disappeared, with the percentage of patients experiencing tinnitus decreasing from 20% to 10% as patients recovered from COVID-19 [6]. This difference may be attributed to the impact of tinnitus severity grading on disease progression and prognosis, as observed in our study. Although more than half of the patients with Grade I tinnitus experienced self-healing from symptoms, more severely affected suffered for a longer duration, leading to a worse situation. Another study involving patients with mild-to-moderate COVID-19 symptoms indicated a lower prevalence of tinnitus, suggesting that the severity of COVID-19 may influence the prevalence and severity grading of post-COVID-19 tinnitus [5]. Furthermore, other studies have emphasized the importance of detailed audiological evaluation in patients who experience tinnitus and hearing loss during and after COVID-19 infection [9, 10].

Assessing the tinnitus severity may help evaluate the severity and progression of post-COVID-19 tinnitus, thereby enabling the implementation of appropriate treatments based on individual patient needs. Various methods have been employed to evaluate the severity of tinnitus, with the THI score being one of the recognized measures [26]. In the general population, approximately 15.3% of patients experience slight tinnitus, 40.8% have mild tinnitus, 28.7% exhibit moderate tinnitus, and 15.2% have severe and catastrophic moderate tinnitus [27]. In the tinnitus severity questionnaire of Biesinger, after COVID-19, the proportion of patients with slight tinnitus (Grade I) increases to 25.3%, surpassing that of the general population. Additionally, 19.4% of patients experience mild tinnitus (Grade II), 22.1% have moderate tinnitus (Grade III), and 33.2% suffer from severe tinnitus (Grade IV). Compared to tinnitus patients in the general population, a relatively higher number of patients experience slight and severe tinnitus after COVID-19.

A study has demonstrated that patients with moderate to severe and catastrophic tinnitus (THI ≥ 38) exhibit distinct cognitive deficits [28]. However, we did not establish a causal relationship between cognition disorder, and we tried to search for a possible association among hearing loss, anxiety, emotion, and tinnitus severity. Anxiety and bad emotions were common psychiatric disorders that occurred during long-term COVID-19 infection, which may interact with tinnitus after COVID-19 infection.

Taking tinnitus severity into consideration may contribute to conjecture and estimate the prognosis for patients with tinnitus after COVID-19. Patients with slight tinnitus have a higher self-healed rate, which decreases significantly as the severity of tinnitus increases. Therefore, it is recommended that patients with severe tinnitus or tinnitus history receive appropriate treatment as soon as possible to prevent long-term damage. However, the cure rate for patients with severe tinnitus after treatment remains lower than that for patients with slight tinnitus.

It is important to acknowledge that this study was conducted by a specialized hospital for eye, ENT conditions, which may have contributed to a higher prevalence of post-COVID-19 tinnitus due to patients seeking treatment for unresolved ENT symptoms. The prevalence rate of post-COVID-19 tinnitus in the general population has been reported to be lower [4, 29], ranging from 0.3% to 20% due to various factors [2–4, 8, 11, 30, 31]. Our study, conducted through an online survey, reported a much higher rate, possibly because the participants were not inpatients or outpatients. Individuals with slight or mild tinnitus may be less likely to seek hospital treatment. Therefore, the actual number of people experiencing post-COVID-19 tinnitus may be greater than currently known. There was also a possibility of gender bias in this study. The incidence of tinnitus among women infected with COVID-19 is high, but there is no significant difference, which may be related to the bias caused by the large number of female respondents (82%). We can investigate and analyse by sex, respectively, to learn the characteristics of tinnitus of respondents of different genders. It is important to recognize that tinnitus symptoms can be challenging for patients to confirm on their own, which may introduce some uncertainty in the accuracy of prognosis due to the lack of objective evaluation criteria. These considerations should be taken into account in future descriptive studies.

## Conclusion

In conclusion, our study highlights that post-COVID-19 tinnitus cases vary in severity, with approximately a quarter of the cases classified as slight. It is encouraging to note that most tinnitus symptoms in this category show spontaneous recovery. However, for patients with more severe tinnitus, the rates of self-healing and recovery are significantly lower. In comparison to patients with slight tinnitus, those with mild tinnitus, and moderate and severe tinnitus had less favourable responses to treatment. In fact, the latter group is more likely to experience no improvement even after treatment, indicating a worse prognosis than patients with slight tinnitus. These findings emphasize the importance of proactive intervention in the management of catastrophic post-COVID-19 tinnitus, particularly in patients who have a higher grade of tinnitus severity.

## Supporting information

Mao et al. supplementary material 1 Mao et al. supplementary material

Mao et al. supplementary material 2 Mao et al. supplementary material

## Data Availability

The datasets generated during or analysed during the current study are enclosed as the supplementary material 2.

## Supplementary material

The supplementary material for this article can be found at http://doi.org/10.1017/S095026882400147X.
