# Supplementary material for: Prevalence and prognosis of tinnitus in post-COVID-19 patients: a cross-sectional survey
Source: Epidemiol Infect. 2024 Nov 8;152:e137. doi: 10.1017/S095026882400147X (PMC11574603; doi:10.1017/S095026882400147X)
Supplement: Mao et al. supplementary material 1 — Mao et al. supplementary material [file S095026882400147Xsup001.docx]

**新冠感染后耳鼻喉症状调查**

**post-COVID-19 Ear, Nose and Throat Symptoms Questionnaire**

尊敬的先生/女士：

Dear Sir or Madam…

您好！为了了解新冠病毒感染后的耳鼻喉科症状，我们邀请您完成此问卷。问卷结果将全部保密，除学术研究外不做其他用途。问卷的填写仅需5分钟，请您认真阅读下面的每一个问题，选择最合适的答案。

Hello! In order to understand the otorhinolaryngology symptoms after COVID-19 infection, we invite you to complete this questionnaire. The results of the questionnaire will be kept confidential and will not be used for other purposes except academic research. It only takes 5 minutes to fill in the questionnaire. Please read each question carefully and choose the most appropriate answer.

复旦大学附属眼耳鼻喉科医院

EYE & ENT Hospital of Fudan University

2023年1月19日

January 19th, 2023

1.姓名_________________________________

1.Name_________________________________

2.性别

2.Sex

○男

○女

○Male

○Female

3.年龄_________________________________

3.Age_________________________________

4.身高（cm）_________________________________

4.Hight（cm）_________________________________

5.体重（kg）_________________________________

5.Weight（kg）_________________________________

6.受教育程度

6.Education level

○小学及以下

○初中

○中专/高中

○大专/大学本科

○硕士研究生

○博士研究生

○Other

○Junior high school

○High school

○Master

○Undergraduate

○Doctor

7.您目前从事的职业_________________________________

7.Your current occupation_________________________________

8.所在地区（选择国外请填具体国家及城市）

8.Location（please specify the country and city if abroad）

○国内

○国外_________________

○China

○Abroad _________________

9.所在城市_________________________________

9.City_________________________________

10.手机号码（非必答，可接受复旦大学附属眼耳鼻喉科医院专业团队联系随访）_________________________________

10.Phone number（optional, used for follow-up by professional team of EYE &ENT Hospital of Fudan Universit）_________________________________

11.新冠感染日期（以最早核酸/抗原阳性时间为准）_________________________________

11.Date of COVID-19 infection (based on the earliest positive nucleic acid/antigen test result) _________________________________

一、耳科症状

一、Ear Symptoms

12.新冠感染后是否出现耳科症状（如：耳聋、耳鸣、耳闷、耳痛、眩晕等）

12.Did you experience any ear-related symptoms after being infected with COVID-19 (e.g. hearing loss, tinnitus, stuffy ears, ear pain, dizziness, etc.)?

○是

○Yes

○否

○No

13.新冠感染后是否出现听力下降

13.Did you experience hearing loss after being infected with COVID-19?

○是

○否（请跳至第15题）

○Yes

○No (skip to question 15)

14.新冠后听力下降出现部位

14.Where did you experience the hearing loss after being infected with COVID-19?

○左耳

○右耳

○双耳

○Left ear

○Right ear

○Both ears

15.新冠感染后是否出现耳鸣

15.Did you experience tinnitus after being infected with COVID-19?

○是

○否（请跳至第22题）

○Yes

○No (skip to question 22)

16.新冠后耳鸣出现部位

16.Where did you experience the tinnitus after being infected with COVID-19?

○左耳

○右耳

○双耳

○Left ear

○Right ear

○Both ears

17.耳鸣声是否影响你的注意力、工作、听他人的声音或享受生活？

17.Is your tinnitus annoying such as affecting your attention, work, ability to hear others or enjoy life?

○是

○有时

○不是

○Yes

○Sometimes

○No

18.耳鸣声是否会让你感到生气、易怒、沮丧、焦虑、没有安全感或让你和家人及朋友关系紧张？

18.Does your tinnitus have major negative consequences on your life such as making you feel angry, irritable, depressed, anxious, insecure, or strain your relationships with family and friends?

○是

○有时

○不是

○Yes

○Sometimes

○No

19.耳鸣声是否让你感到绝望、无法控制、不能忍受或者觉得自己患了很严重的疾病？

19.Are you able to work? Can you do your housework? Can you take care of your family and not feeling hopeless, out of control, intolerable, or make you think you have a serious illness?

○是

○有时

○不是

○Yes

○Sometimes

○No

20.耳鸣声是否影响你的睡眠？（如出现入睡困难、夜间觉醒、早醒等症状）

20.Did the tinnitus affect your sleep? (e.g. difficulty falling asleep, waking up at night, early awakening, etc.)

○没有影响

○轻微影响

○显著影响

○严重影响或没有睡觉

○No effect

○Slight impact

○Significant impact

○Severe impact or no sleep

21.总睡眠质量

21.Did you satisfied with your quality of overall sleep？

○满意

○轻微不满

○显著不满

○严重不满或没有睡觉

○Satisfied

○Slightly dissatisfied

○Significantly dissatisfied

○Very dissatisfied or couldn’t sleep

22.新冠感染后是否出现耳闷

22.Did you experience ear fullness after being infected with COVID-19？

○是

○否（请跳至第24题）

○Yes

○No (skip to question 24)

23.新冠后耳闷出现部位

23.Where did you experience ear fullness after COVID-19 infection？

○左耳

○右耳

○双耳

○Left ear

○Right ear

○Both ears

24.新冠感染后是否出现耳痛

24.Did you experience ear pain after being infected with COVID-19?

○是

○否（请跳至第26题）

○Yes

○No (skip to question 26)

25.新冠后耳痛出现部位

25.Where did you experience ear pain after COVID-19 infection？

○左耳

○右耳

○双耳

○Left ear

○Right ear

○Both ears

26.新冠感染后是否出现眩晕（天旋地转）

26.Did you experience dizziness (vertigo) after being infected with COVID-19?

○是

○否

○Yes

○No

27.新冠感染后出现耳科症状就诊情况

27.Did you see a doctor because of the ear symptoms after COVID-19 infection?

○是

○否

○Yes

○No

28.新冠感染后耳科症状对日常生活的影响程度（0表示没有影响，10表示影响极大）

28.Level of impact on daily life due to ear symptoms after COVID-19 infection (0 means no impact, 10 means extremely impactful)

|  | 0 | 1 | 2 | 3 | 4 | 5 | 6 | 7 | 8 | 9 | 10 |
| --- | --- | --- | --- | --- | --- | --- | --- | --- | --- | --- | --- |
| 听力下降 | ○ | ○ | ○ | ○ | ○ | ○ | ○ | ○ | ○ | ○ | ○ |
| 耳鸣 | ○ | ○ | ○ | ○ | ○ | ○ | ○ | ○ | ○ | ○ | ○ |
| 耳闷 | ○ | ○ | ○ | ○ | ○ | ○ | ○ | ○ | ○ | ○ | ○ |
| 耳痛 | ○ | ○ | ○ | ○ | ○ | ○ | ○ | ○ | ○ | ○ | ○ |
| 眩晕 | ○ | ○ | ○ | ○ | ○ | ○ | ○ | ○ | ○ | ○ | ○ |

|  | 0 | 1 | 2 | 3 | 4 | 5 | 6 | 7 | 8 | 9 | 10 |
| --- | --- | --- | --- | --- | --- | --- | --- | --- | --- | --- | --- |
| Hearing loss | ○ | ○ | ○ | ○ | ○ | ○ | ○ | ○ | ○ | ○ | ○ |
| Tinnitus | ○ | ○ | ○ | ○ | ○ | ○ | ○ | ○ | ○ | ○ | ○ |
| Ear fullness | ○ | ○ | ○ | ○ | ○ | ○ | ○ | ○ | ○ | ○ | ○ |
| Earache | ○ | ○ | ○ | ○ | ○ | ○ | ○ | ○ | ○ | ○ | ○ |
| Dizziness | ○ | ○ | ○ | ○ | ○ | ○ | ○ | ○ | ○ | ○ | ○ |

29.新冠感染后出现耳科症状转归情况

29.Prognosis of ear symptoms after COVID-19 infection

|  | 无 | 未治疗，自愈 | 治疗后完全消失 | 治疗后部分症状消失 | 治疗后无明显好转 |
| --- | --- | --- | --- | --- | --- |
| 听力下降 | ○ | ○ | ○ | ○ | ○ |
| 耳鸣 | ○ | ○ | ○ | ○ | ○ |
| 耳闷 | ○ | ○ | ○ | ○ | ○ |
| 耳痛 | ○ | ○ | ○ | ○ | ○ |
| 眩晕 | ○ | ○ | ○ | ○ | ○ |

|  | No | Self-healed | Full remission | Relief | Failure |
| --- | --- | --- | --- | --- | --- |
| Hearing loss | ○ | ○ | ○ | ○ | ○ |
| Tinnitus | ○ | ○ | ○ | ○ | ○ |
| Ear fullness | ○ | ○ | ○ | ○ | ○ |
| Earache | ○ | ○ | ○ | ○ | ○ |
| Dizziness | ○ | ○ | ○ | ○ | ○ |

二、鼻科症状

二、Nose Symptoms

30.新冠感染后是否出现鼻科症状（如：鼻塞、流涕、嗅觉减退等）

30.Did you experience nose symptoms after being infected with COVID-19? (e.g. nasal obstruction, rhinorrhea, hyposmia, etc.)

○是

○否

○Yes

○No

31.新冠感染后是否出现鼻塞

31.Did you experience nasal obstruction after being infected with COVID-19?

○是

○否

○Yes

○No

32.新冠感染后是否出现流涕

32.Did you experience rhinorrhea after being infected with COVID-19?

○是

○否

○Yes

○No

33.新冠感染后是否出现嗅觉减退

33.Did you experience hyposmia after being infected with COVID-19?

○是

○否

○Yes

○No

34.新冠感染后鼻科症状对日常生活的影响程度（0表示没有影响，10表示影响极大）

34.Level of impact on daily life due to nose symptoms after COVID-19 infection (0 means no impact, 10 means extremely impactful)

|  | 0 | 1 | 2 | 3 | 4 | 5 | 6 | 7 | 8 | 9 | 10 |
| --- | --- | --- | --- | --- | --- | --- | --- | --- | --- | --- | --- |
| 鼻塞 | ○ | ○ | ○ | ○ | ○ | ○ | ○ | ○ | ○ | ○ | ○ |
| 流涕 | ○ | ○ | ○ | ○ | ○ | ○ | ○ | ○ | ○ | ○ | ○ |
| 嗅觉减退 | ○ | ○ | ○ | ○ | ○ | ○ | ○ | ○ | ○ | ○ | ○ |

|  | 0 | 1 | 2 | 3 | 4 | 5 | 6 | 7 | 8 | 9 | 10 |
| --- | --- | --- | --- | --- | --- | --- | --- | --- | --- | --- | --- |
| Nasal obstruction | ○ | ○ | ○ | ○ | ○ | ○ | ○ | ○ | ○ | ○ | ○ |
| rhinorrhea | ○ | ○ | ○ | ○ | ○ | ○ | ○ | ○ | ○ | ○ | ○ |
| hyposmia | ○ | ○ | ○ | ○ | ○ | ○ | ○ | ○ | ○ | ○ | ○ |

35.新冠感染后出现鼻科症状就诊情况

35.Did you see a doctor because of the nose symptoms after COVID-19 infection?

○是

○否

○Yes

○No

36.新冠感染后出现鼻科症状转归情况

36.Prognosis of nose symptoms after COVID-19 infection

|  | 无 | 未治疗，自愈 | 治疗后完全消失 | 治疗后部分症状消失 | 残留症状 |
| --- | --- | --- | --- | --- | --- |
| 鼻塞 | ○ | ○ | ○ | ○ | ○ |
| 流涕 | ○ | ○ | ○ | ○ | ○ |
| 嗅觉减退 | ○ | ○ | ○ | ○ | ○ |

|  | No | Self-healed | Full remission | Relief | Failure |
| --- | --- | --- | --- | --- | --- |
| Nasal obstruction | ○ | ○ | ○ | ○ | ○ |
| Rhinorrhea | ○ | ○ | ○ | ○ | ○ |
| Hyposmia | ○ | ○ | ○ | ○ | ○ |

三、咽喉症状

三、Throat Symptoms

37. 新冠感染后是否出现咽喉症状（如：咳嗽、咽痛、味觉减退等）

Did you experience throat symptoms after being infected with COVID-19? (e.g. cough, pharyngalgia, hypogeusia, etc.)

○是

○否

○Yes

○No

38.新冠感染后是否出现咳嗽

38.Did you experience cough after being infected with COVID-19?

○是

○否

○Yes

○No

39.新冠感染后是否出现咽痛

39.Did you experience pharyngalgia after being infected with COVID-19?

○是

○否

○Yes

○No

40.新冠感染后是否出现味觉减退

40.Did you experience hypogeusia after being infected with COVID-19?

○是

○否

○Yes

○No

41.新冠后咽喉症状对日常生活的影响程度（0表示没有影响，10表示影响极大）

41.Level of impact on daily life due to throat symptoms after COVID-19 infection (0 means no impact, 10 means extremely impactful)

|  | 0 | 1 | 2 | 3 | 4 | 5 | 6 | 7 | 8 | 9 | 10 |
| --- | --- | --- | --- | --- | --- | --- | --- | --- | --- | --- | --- |
| 咳嗽 | ○ | ○ | ○ | ○ | ○ | ○ | ○ | ○ | ○ | ○ | ○ |
| 咽痛 | ○ | ○ | ○ | ○ | ○ | ○ | ○ | ○ | ○ | ○ | ○ |
| 味觉减退 | ○ | ○ | ○ | ○ | ○ | ○ | ○ | ○ | ○ | ○ | ○ |

|  | 0 | 1 | 2 | 3 | 4 | 5 | 6 | 7 | 8 | 9 | 10 |
| --- | --- | --- | --- | --- | --- | --- | --- | --- | --- | --- | --- |
| cough | ○ | ○ | ○ | ○ | ○ | ○ | ○ | ○ | ○ | ○ | ○ |
| pharyngalgia | ○ | ○ | ○ | ○ | ○ | ○ | ○ | ○ | ○ | ○ | ○ |
| hypogeusia | ○ | ○ | ○ | ○ | ○ | ○ | ○ | ○ | ○ | ○ | ○ |

42.新冠感染后出现咽喉症状就诊情况

42.Did you see a doctor because of the throat symptoms after COVID-19 infection?

○是

○否

○Yes

○No

43.新冠感染后出现咽喉症状转归情况

43.Prognosis of nose symptoms after COVID-19 infection

|  | 无 | 未治疗，自愈 | 治疗后完全消失 | 治疗后部分症状消失 | 残留症状 |
| --- | --- | --- | --- | --- | --- |
| 咳嗽 | ○ | ○ | ○ | ○ | ○ |
| 咽痛 | ○ | ○ | ○ | ○ | ○ |
| 味觉减退 | ○ | ○ | ○ | ○ | ○ |

|  | No | Self-healed | Full remission | Relief | Failure |
| --- | --- | --- | --- | --- | --- |
| Cough | ○ | ○ | ○ | ○ | ○ |
| Pharyngalgia | ○ | ○ | ○ | ○ | ○ |
| Hypogeusia | ○ | ○ | ○ | ○ | ○ |

四、认知和情感

四、Cognitive and Emotional State after COVID-19 infection

44.新冠感染后的认知状态

44.Cognitive State after COVID-19 Infection

○能够记住大多数事情，思路清晰，并且能解决每天的问难

○能够记住大多数事情，但想事情和解决每天的问题时有点困难

○有点健忘，但是思路清晰，能解决每天的问题

○有点健忘，想事情和解决每天的问题时有点困难

○非常健忘，想事情和解决每天问题有极大的困难

○什么事情也记不起来，不能想事情和解决每天问题

○Able to remember most things, clear-minded, and able to solve daily problems.

○Able to remember most things, but have some difficulty thinking and solving daily problems.

○Somewhat forgetful, but clear-minded and able to solve daily problems.

○Somewhat forgetful and have some difficulty thinking and solving daily problems.

○Very forgetful and have great difficulty thinking and solving daily problems.

○Unable to remember anything and unable to think and solve daily problems.

45.新冠感染后的情感状态

45.Emotional State after COVID-19 Infection

○幸福，对生命充满兴趣

○有点幸福

○有点不幸福

○非常不幸福

○如此不幸福，以致于觉得生命没有价值

○Happy and interested in life

○Somewhat happy

○Somewhat unhappy

○Very unhappy

○So unhappy to the point of feeling life has no valu

46.在过去的两周里，感觉紧张、焦虑或烦躁的频率有多少？

46.In the past two weeks, how often have you felt nervous, anxious, or restless? *

○完全不会

○几天

○一半以上的日子

○几乎每天

○Not at all

○A few days

○More than half of the days

○Almost every day

47.在过去的两周里，不能停止或控制担忧的频率有多少？

47.In the past two weeks, how often have you been unable to stop or control worrying *

○完全不会

○几天

○一半以上的日子

○几乎每天

○Not at all

○A few days

○More than half of the days

○Almost every day

48.在过去的两周里，做事时提不起劲或没有兴趣的频率有多少？

48.In the past two weeks, how often have you felt a lack of energy or interest in doing things?

○完全不会

○几天

○一半以上的日子

○几乎每天

○Not at all

○A few days

○More than half of the days

○Almost every day

49.在过去的两周里，感到心情低落、沮丧或绝望的频率有多少？

49.In the past two weeks, how often have you felt down, depressed, or hopeless?

○完全不会

○几天

○一半以上的日子

○几乎每天

○Not at all

○A few days

○More than half of the days

○Almost every day

五、既往病史

五、Medical History

50.既往耳鼻喉科病史（如耳鸣、中耳炎、鼻炎、咽炎等，若无则填“无”） _________________________________

50.Do you haven ear, nose, and throat diseases such as tinnitus, otitis media, rhinitis, pharyngitis, etc. If none, please write "none" _________________________________

51.是否患有高血压（若是，填写患病时间，如“5年”）

51.Do you have hypertension? (If yes, please write the duration of the disease, such as "5 years") *

○是_________

○否

○Yes_________

○No

52.是否患有糖尿病（若是，填写患病时间，如“5年”）

52.Do you have diabetes? (If yes, please write the duration of the disease, such as "5 years")

○是_________

○否

○Yes_________

○No
